# A visual imagery paradigm for BCI strategies using imagined flickering patterns

**DOI:** 10.1038/s41598-026-41324-6

**Published:** 2026-03-04

**Authors:** Simone Priori, Paolo Ricci, Davide Consoli, Arturo Micheli, Adrien Merlini, Francesco P. Andriulli

**Affiliations:** 1 Department of Electronics and Telecommunication, Politecnico di Torino, 10129 Torino, Italy; 2 OpenGeoHub Foundation, 6865 HK Doorwerth, Netherlands; 3 Microwave Department, IMT Atlantique, 29238 Brest, France

**Keywords:** Neuroscience, Psychology, Psychology

## Abstract

Steady state visually evoked potentials (SSVEPs) are a popular type of control signals in brain-computer interfaces (BCIs), in which they are typically elicited by observing a visual stimulus flashing at a specific frequency. For some patients, using SSVEP as control signal for a BCI can be difficult, for instance if they are unable to focus their gaze over the visual stimuli. To address this issue, some approaches were presented to design a gaze-independent SSVEP-controlled BCI but some difficulties have been reported, for instance for patients suffering from locked-in syndrome. In this work we employ a visual imagery (VI) signal, in which the visual stimulus is imagined instead of observed, to drive a BCI system and offer an alternative for patients that encounter issues with standard SSVEP approaches. We tested the proposed approach with 20 untrained subjects within a 3-classes BCI resulting in an offline classification accuracy of 60.93%. These results demonstrate how this gaze-independent BCI can be used by inexperienced BCI users.

## Introduction

A brain-computer interface (BCI) allows a user to interact with an electronic device using only their brain activity^1,2^. Patients suffering from neurodegenerative diseases or from the after-effects of traumatic events can greatly benefit from BCIs, even in the initial phase of rehabilitation, by facilitating their interaction with the environment^3–5^. BCIs can be classified depending on the type of driving signal they rely on and on the method chosen to record it^6^. Thanks to its limited cost and high temporal resolution, the electroencephalography (EEG) is one of the most widespread recording systems^7^.

Between all possible strategies in EEG-based BCI applications, steady state visually evoked potentials (SSVEPs) are well-known driving signals and have been widely studied and successfully applied^8–13^. The wide adoption of SSVEPs is in part due to the fact that they can be elicited and recognized with relative ease: a light pattern flashing at a specific frequency results in a signal characterized by the presence of electrical activity in the brain at the frequency of stimulation and its harmonics^14^. This signal is usually recorded over the occipital lobe.

However, there are inherent limitations to SSVEP techniques following from the need for an external apparatus to elicit the desired response in the subject. In particular, SSVEP-based applications perform best with subjects that are able to gaze accurately and in a consistent manner at a specific visual stimulus, e.g a pattern projected on a screen, and their accuracy drops significantly if the subject has visual or ocular motility impairments^15^. As a result, the usage of SSVEP-driven BCI can prove challenging for certain subjects that suffer from pathologies like the locked-in syndrome or post-ictus patients. To improve the usability of SSVEP-based BCIs for subjects that have a reduced ability to control their gaze, attempts have been made to develop a gaze-independent system, but those subjects still experienced significantly lower performances when compared to healthy subjects^16^. Further steps were taken to develop different non-invasive BCIs for patients with severe motor impairment, such as auditory and visual P300 systems^17–19^. The visual P300 shares some of the limitations of SSVEPs and has been shown to exhibit consistently lower accuracy^18^ and the auditory P300 has been reported to be challenging to work with^17^. Another class of alternative approaches relies on invasive modalities that however have inherent risks, in particular for patients suffering from brain injuries^20^.

Another way to overcome some of the limitations of SSVEPs is to change the driving signal from an evoked to a visually imagined signal. Visual imagery (VI) is the act of producing the representation of a visual event without the corresponding input associated to visual perception (VP) and has been described by Pearson et al.^21^ as a “weak form of visual perception”. Approaches using VI as the driving signal for BCI applications have been presented^22^. In these works the users typically imagine static^23–25^ or moving images^26,27^, ranging from simple characters and letters to faces and photos. Studies show that VI and VP activity share the activation of some of the networks^28,29^. For instance, the eye pupil have been reported to adjust to the imagined light even without changes in the environmental luminosity^30–32^. Similarly, subjects imagining the stimuli generating the McColloguh effect have been reported to experience the same optical illusion as when they are shown the stimuli^34–36^. A similar effect has been reported when imagining the visual stimuli that creates the SSVEP response in an SSVEP-based BCI system^33^, which showed the possibility to translate the architecture used for a 3-classes SSVEP driven BCI to a 3-classes VI driven BCI. A following, independent study^37^ showed compatible results with those of^33^ even though a different approach was taken, for instance by letting the subjects imagine only one frequency during each experimental session. A series of 12 VI sessions were executed in Saichoo et al.^37^, during which the subjects imagined flashing patterns at different frequencies, specifically 7Hz, 8Hz, 11Hz 13Hz. The subjects had a specific frequency to imagine during each of these 12 sessions, following a predetermined sequence that was the same for all of them. During the experiment the activity consisted of comparing one imagined frequency against the rest state.

In this work, we explore the possibility of designing a BCI system that is driven by a signal corresponding to an imagined visual stimulus. Because of the similarities between the VP and VI signal we can then use the characteristics of SSVEP signals to have a set of distinguishable VI signal. This strategy allows for a system that shares much of the structure of an SSVEP-driven BCI while removing the constraints on the patient’s gaze. The experimental setup for the present work builds upon the one presented in^33^, in this preliminary work a series of offline experiments were conducted on a single subject, consisting of separate VI, SSVEP or mixed activities between two frequencies, 5Hz, 7Hz. The work of Micheli et al.^33^ is expanded in the current experiment, involving multiple people, different frequencies and an online analysis. To validate the scheme we conducted experiments on 20 subjects that were initially asked to familiarize themselves with visual stimuli in the form of checkerboard patterns flickering at specific frequencies (5Hz, 7Hz, 9Hz, 12Hz) that are commonplace in SSVEP-based BCIs. The visual stimuli were then removed after the subjects were accustomed to them, and the subjects were asked to imagine the previously shown flickering patterns for the following steps of the experiment, to control the BCI system.

The remainder of this paper is organized as follows: the proposed BCI system and its setup are described in Sect. 2 while the results of the experiments are delineated in Sect. 3 and interpreted in Sect. 4. Concluding remarks are then given in Sect. 5.

## Method

### Proposed BCI paradigm

The proposed BCI paradigm is a 3-classes VI-driven BCI corresponding to a rest state and two distinct VI states triggered by the users imagining a pattern flickering at two different frequencies. The pattern to be imagined in both VI classes is a checkerboard and only the frequency of flickering will change. The determination of these frequencies is partially subject-specific and is part of the initial setup with each subject.

### Participants

20 subjects, aged 23–31, 5 females and 15 males, took part in this study. None of the subjects involved with the experiment have been diagnosed with epilepsy previously to the experiments. This study with human participants was approved by the “Comitato Etico per la Ricerca del Politecnico di Torino (CER-Polito)” under the Ethical Declarations of Helsinki, Council for International Organizations of Medical Sciences, and the World Health Organization guidelines with protocol number 70562/2024. The subjects participation was always voluntary and the subjects were all fully informed and provided with documentation on the experiment before giving their consent. The signed consent forms are stored confidentially. To anonymize the data all 20 subjects that participated in the experiment are designated internally by a 6 digit number created randomly. In this paper the subjects will be referred to by number, from 1 to 20, this number is not representative of the actual order of execution of the experiment. The subject of Fig. 4 is one of the authors of this paper. No data used on this paper was recorded on him and the figure simply showcases the setup.

### Driving signal

**Fig. 1:**
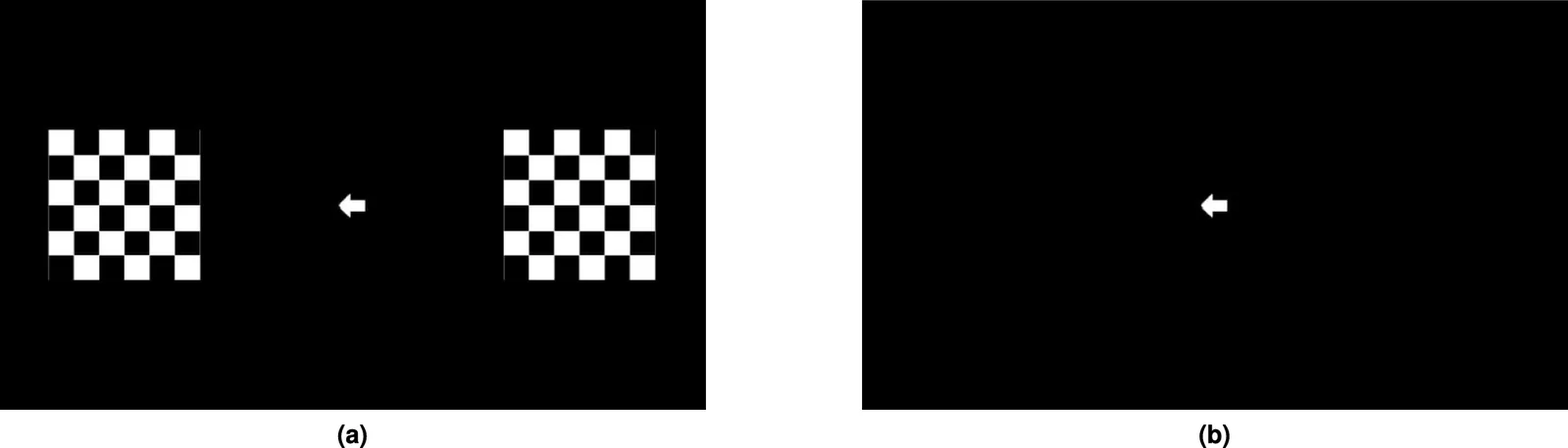
Example of screens presented to the user. **a** Checkerboard patterns flashing during the task, shown as presented in *Step 2*. During *Step 1* there are two other checkerboards, one above and one below the arrow. **b** Arrow pointed at the VI task to execute, shown as it is presented from *Step 2* to *Step 4*.

The driving VI signal for the set of experiments is an imagined flickering pattern that would elicit an SSVEP response if it were actually shown to the subjects. In this work the pattern is a flickering checkerboard, as illustrated in Fig. 1.

**Fig. 2:**
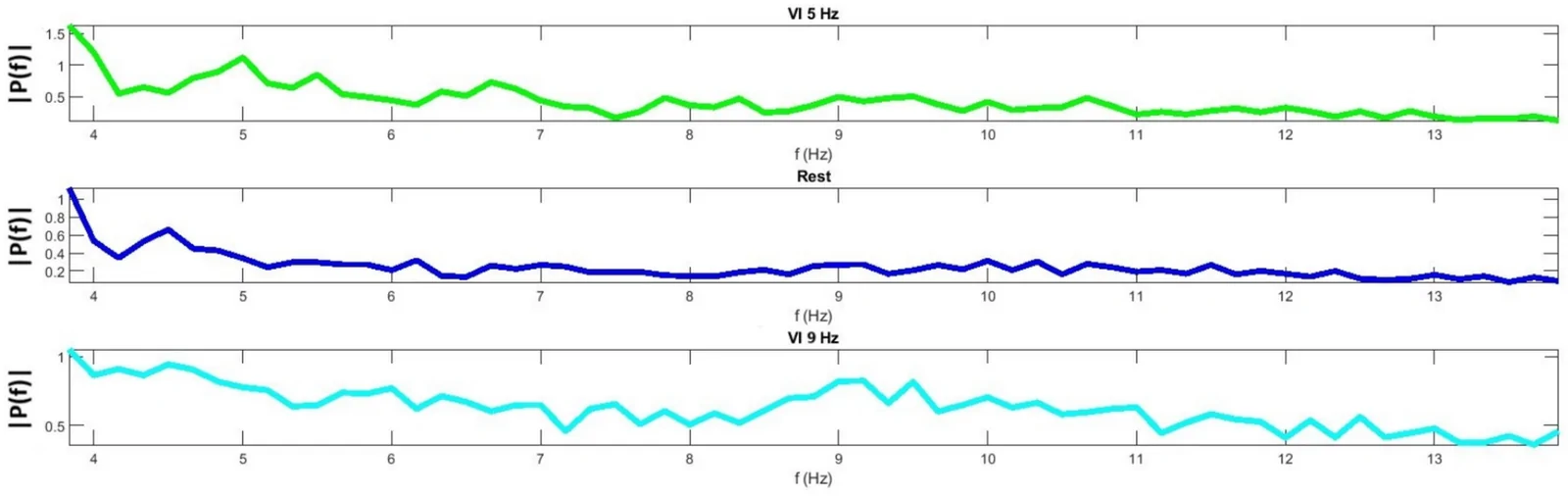
signal recorded over the occipital cortex during a VI experiment on S.18. The peaks in the PSD track align with similar recordings obtainable in SSVEP experiments, although the peaks at higher frequency are not clearly present.

During VI, the power spectral density (PSD) of the signal recorded over the occipital cortex will display strong similarities with the corresponding VP stimulus, with the characteristic presence of a series of power spikes over the stimulation frequency and its harmonics^33,37^. To illustrate this, some VI and SSVEP PSDs—acquired as part of this work’s experiments—are presented in Fig. 2.

The imagery signal has similarities with the corresponding evoked potential signal with peaks spread around the imagined frequencies. Brain activity during VI tasks has been studied in numerous works^28,29,38–41^ . In particular the review works^28,29^ studied the brain networks shared between visual perception and imagination, with Dijkstra et al.^29^ comparing it for healthy subjects and Kaski et al.^28^ doing so for subjects with injuries in the occipital area. Goldenberg et al.^38,39^ monitored the blood flow for subject imagining images and how it varies increasing the complexity of the task, similarly Charlot et al.^41^ compared the blood flow during different cognitive tasks, specifically verb conjugation and VI. Farah et al.^40^ studied the EEG recordings of subjects asked to produce a mental image of something they read, comparing it to the recordings obtained by the same subjects while reading it. The results obtained from these studies show that the brain areas involved with VI include the frontal, temporal, occipital and parietal lobe.^28,29,38–41^

Because VI and VP share neural activation areas^28,29,36,38–41^, the areas monitored for VI activity in this study are the occipital, parietal, and frontal lobe^25^ with emphasis on its rightmost portion^27^.

### Acquisition

**Fig. 3:**
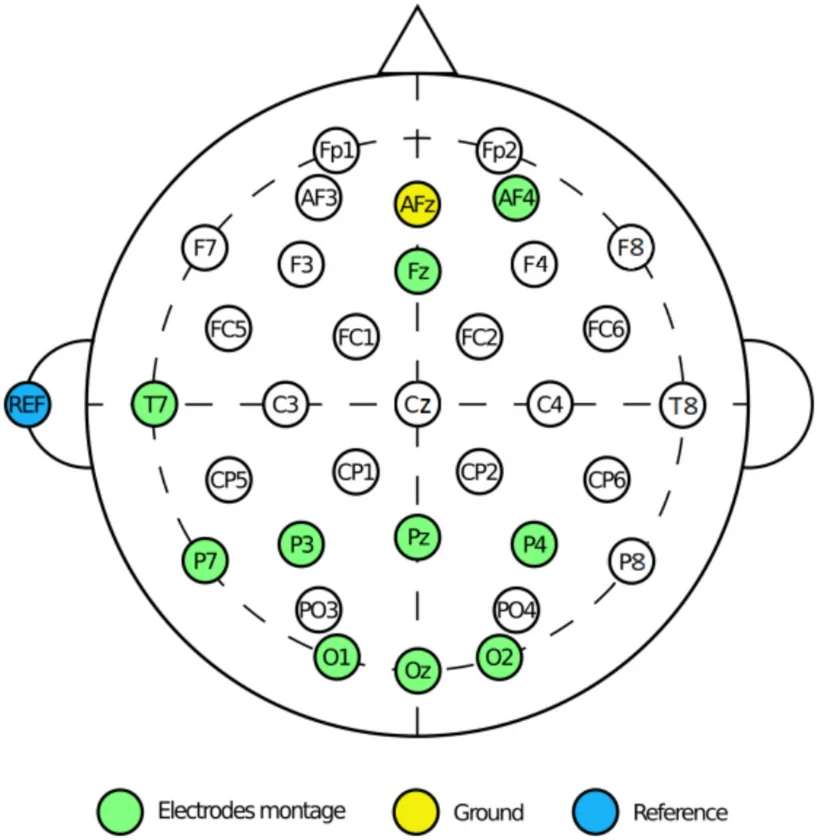
Electrode placement used for the EEG signal acquisition.

The brain activity was recorded using the g.SCARABEO (g.tec) EEG wet active electrodes and g.GAMMAgel (g.tec). During the experiment a montage of 12 electrodes was used, including the ground and reference, as shown in Fig. 3.

**Fig. 4:**
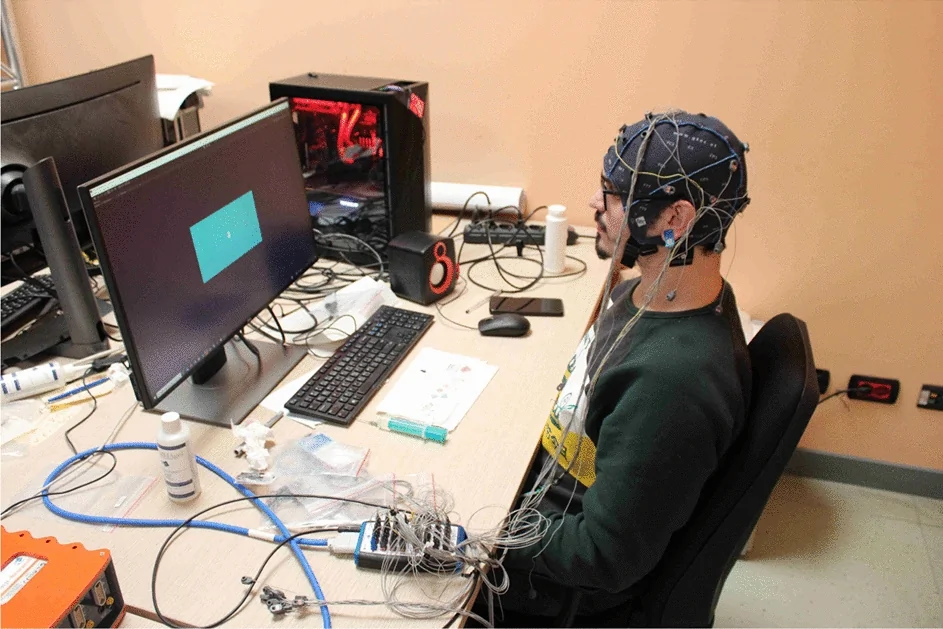
Photography of the experimental setup and subject position during the experiment. On the screen it is shown the command for the rest state, a blue rectangle in the middle of the screen.

The 10 electrodes considered in the classification are [O2, O1, Pz, P3, P4, Oz, T7, P7, AF4, F8]. The electrode placement is similar to the one that was used in the VI experiment in^33^. The electrode array is held together with the EEG cap g.GAMMAcap (g.tec) and all the electrodes are connected to a g.HIamp-Research amplifier through a 64-channels electrode connector box. The amplifier is connected to a computer with Matlab installed. The devices interact thanks to a Simulink model. The commands for all the steps of the experiment are given to the subjects through a Unity application that shows them what is the next task they should execute. All experiments were performed in a quiet room with standard lighting; the subjects sat on an office chair at 60cm from the computer screen, as shown in Fig. 4.

During the experiment particular care was taken to avoid external distractions for the subjects. After each session, the subjects were asked about their condition to ensure the absence of possible headaches or other discomforts which none of the subjects reported.

### Recordings

A standardized experimental protocol composed of four steps was applied to each of the 20 subjects. A high level description of this steps is given here, while the details of their implementation are provided later in the chapter, for readability.

*Step 1: Selection of patient-specific VI frequencies.* To select a subject-specific pair of frequencies, that the subjects will have to imagine during the actual VI-BCI phase, all subjects go through a first SSVEP session with 4 checkered patterns flickering at 5Hz, 7Hz, 9Hz, 12Hz, respectively, corresponding to 4 SSVEP classes to which a rest state is added. The subjects have to fixate their gaze on one of the flickering patterns following on-screen instructions. This recording is then used to select the two frequencies that will be used for the rest of the experiment. Each class has 15 repetitions lasting 3 seconds each.

*Step 2: Mixed SSVEP/VI training session.* To help the subjects getting accustomed to the selected frequencies that they will need to imagine for the rest of the experiment, they go through a training session comprising multiple recordings with visual imagery tasks, SSVEP stimulation, and rest. Only the two selected frequencies are used. The SSVEP tasks are done in the same manner as *Step 1*. The subjects have to imagine the flickering pattern seen in the SSVEP tasks during the VI ones. As before, the sequence is communicated to the subject through on-screen instructions. Each class has 18 repetitions and the process is repeated 3 times.

*Step 3: Offline VI session.* The subjects execute the offline portion of the experiment, comprised of 10 recordings with only VI tasks and rest (the SSVEP stimulus is no longer presented to the user). During the VI tasks the subject has to remember and imagine the flickering patterns used in the previous step. Each class has 20 repetitions lasting 9 seconds each.

*Step 4: Online VI session.* The online VI session consists of a single recording with only VI tasks and rest. Each class has 20 repetitions lasting 5 seconds each.

In all steps, the subjects were instructed to rest for 2 minutes between each recording and the task to be executed was indicated by an arrow (see Fig. 1b) or by a blue square for the rest. During the SSVEP tasks, one flickering checkerboard per frequency is shown and the arrow points towards the flickering square to concentrate on. In *Step 1* four checkerboards were present on the screen during SSVEP tasks. In *Step 2*, only two checkerboards were present, as shown in Fig. 1. For the VI tasks in *Step 2*, *Step 3*, and *Step 4*, the direction indicated by the arrow informs the subject on which frequency to imagine without any flickering visual clue (see Fig. 1b), and the lower frequency is always placed on the left of the screen. An auditory feedback informs the subject when a tasks terminates. During the first three steps, the task sequence is selected randomly for each subjects and the specific sequence of tasks in each recording is not disclosed to them before the execution. For step 4, the sequence is the same for all subjects but neither this information nor the specific sequence is disclosed to the subjects before the start of the online experiment. The sequence of events for the online experiment consisted in 10 events for each class ordered as: “lower frequency”, “rest”, “higher frequency”, and then repeated once.

To avoid considering signals recorded during the change of a task, only a portion of the recorded signals was taken into consideration. For *Step 1*, a 1-second portion of the signal was used for each task, from 1.8 to 2.8 s. Such an interval helps excluding signal related to the eye muscles twitching and is coherent with the response time for the SSVEP signal, that has been estimated to be of around 250 ms^46^. In *Step 3* and *Step 4*, a 6-seconds interval from 1 to 7 s and a 3-seconds interval from 1 to 4 s were considered, respectively.

The recorded data was processed through the amplifier and Matlab with a series of filters:

- a 60 Hz low-pass filter used to reduce the effect of electromyographic signals and high frequency electromagnetic noise;
- a 48–52 Hz notch filter: used to reject the line noise;
- a 3–36 Hz band-pass filter: specifically an 8th order Butterworth filter.

The first two were applied by the recorder during the task, the third filter was applied afterwards. The data processed through all the filters was then converted in a vector comprised of the average power spectral density (PSD) from 3 to 36 Hz. This was done separately for every trial and every electrode. During *Step 3* and *Step 4*, these vectors were the features used by the classifier. The PSD was evaluated with a Welch periodogram, with the signal divided in intervals of 2 seconds with an Hamming window and 50% overlap.

It should be noted that the training procedure proposed here is not intrinsically gaze-independent, which is not needed for this study since all subjects were healthy. However, in a practical application with patients the training can be easily made gaze-independent simply by showing only one flickering image at a time during *Step 1* and the SSVEP portion of *Step 2*.

### Frequency selection

We opted not to use the same couple of frequencies for all the subjects, because previous experiences indicated that the subjects are not equally responsive to the same frequencies in SSVEP experiments. Some subjects have been reported to show preferences for stimuli at higher or lower frequencies for SSVEP stimulation^42^. To standardize the frequency selection process, each of the four possible frequencies (5 Hz, 7 Hz, 9 Hz, 12 Hz) is assigned a numerical score based on the user’s performance in an SSVEP experiment, and the two frequencies with the highest scores are selected for that user. The target frequencies are selected to avoid conflicts between the second harmonic of the lower frequencies and the first harmonic of the higher frequencies The numerical score is assigned following a technique inspired by the “Empirical mode decomposition and power spectrum peaks analysis” method to detect SSVEP signals^43^. The specific data that is used for the scoring is the data recorded in *Step 1* over the occipital area of the subjects, specifically Oz, O1 and O2, during an SSVEP recording. The numerical score for the frequencies is computed as follows: the data is divided into each separate task, the 4 flickering patterns and the rest, and the PSD is then evaluated and scored for each repetition separately. The score is evaluated from the amplitude of the PSD peaks, defined as

1 $$\begin{aligned} \Delta p_{\pm }(f)=M_{\pm }(f)-m_{\pm }(f) \end{aligned}$$

where $M_{\pm }(f)$ is the maximum of the PSD in the close neighborhood of the peak ($\pm 0.05\,\hbox {Hz}$) and $m_{\pm }$ is the minimum of the PSD in a larger neighborhood of the peak ($\pm 0.5\,\hbox {Hz}$). This operation is done separately for the left and the right side of the peak, indicated by $\Delta p_{-}(f)$ or $\Delta p_{+}(f)$, with $\Delta p_{-}(f)$ calculated on the left side interval and $\Delta p_{+}(f)$ on the right side interval. The score *S* is then computed as

2 $$\begin{aligned} S=\max (\Delta p_{+}(f),\Delta p_{-}(f)) / \iota . \end{aligned}$$

where *ι* is the width of the peak at half of its height. For each task, this process is repeated for the PSD peak at the frequency of stimulation and its second harmonic, the two values obtained for these two peaks are then summed. The values obtained for each frequency in each repetition are then summed. The two frequencies with the highest score are then selected as the optimal or “primary” frequencies and used in all further measures for the subject.

After the frequency selection all subject will be assigned a single frequency couple, from the 4 initial frequencies, that will be used for the remainder of the experiment.

### Classifier

The data of the VI experiment is classified with a support vector machine (SVM) with a linear kernel. The SVM is used as a 3-classes classifier, distinguishing between the two VI classes and the rest. This technique has proven to be performing for SSVEP applications^44,45^, and in previous VI experiments^33^.

The classifiers used for each subject were trained on 8 of their 10 recordings. The data was taken from the 10 recording sessions executed by the subject. The 2 remaining recordings were used as the test set. A 5-fold temporal cross-validation was applied. In each iteration 2 of the 8 recordings of the classifiers were swapped with the test set.

## Results

### Frequency selection and evaluation of the process

We will refer to the frequencies selected for each subject as primary frequencies The frequency selection procedure selected as primary frequencies the couple $(5\,\hbox {Hz}, 9\,\hbox {Hz})$ for the majority (11/20) of the subjects. The frequency couple $(5\,\hbox {Hz}, 12\,\hbox {Hz})$ was selected for 4 of the remaining subjects, $(9\,\hbox {Hz}, 12\,\hbox {Hz})$ for 3 subjects, and $(5\,\hbox {Hz}, 7\,\hbox {Hz})$ for 2 subjects. There was no subject for which the process indicated $(7\,\hbox {Hz}, 12\,\hbox {Hz})$ or $(7\,\hbox {Hz}, 9\,\hbox {Hz})$. These results are summarized in Table 1.

**Table 1:** Frequency couples selected as primary frequencies for each subject in the experiment.

Primary frequencies	Subjects
$(5\,\hbox {Hz}, 9\,\hbox {Hz})$	s.1 s.2 s.6 s.7
	s.10 s.12 s.15 s.17
	s.18 s.19 s.20
$(5\,\hbox {Hz}, 12\,\hbox {Hz})$	s.3 s.8 s.9 s.11
$(9\,\hbox {Hz}, 12\,\hbox {Hz})$	s.5 s.13 s.16
$(5\,\hbox {Hz}, 7\,\hbox {Hz})$	s.4 s.14

**Fig. 5:**
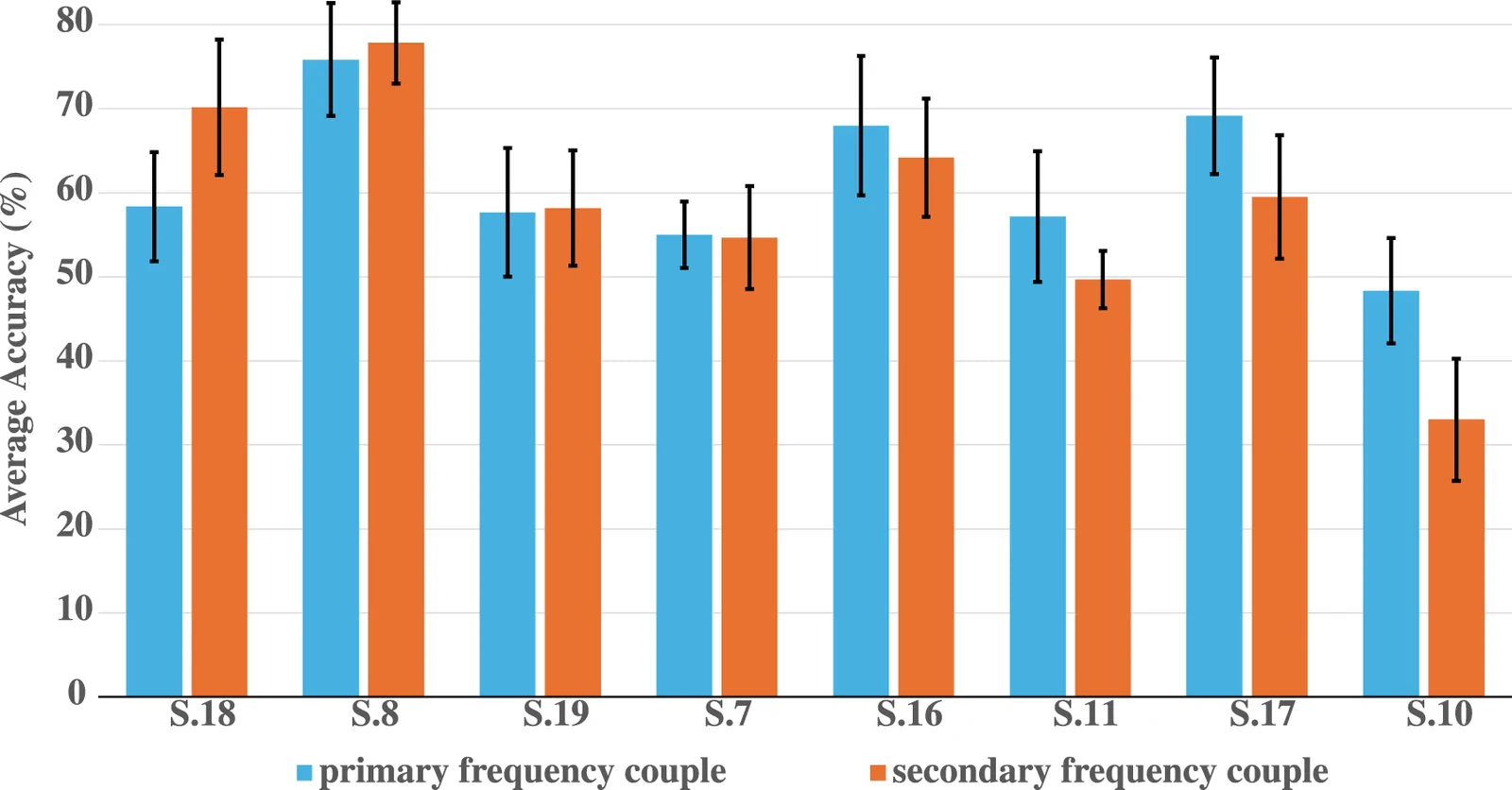
Comparison between the average accuracies obtained on the primary and secondary frequency couple for the 8 subjects that agreed to repeat the experiment. Ordered in the same way as Table 2.

To verify the robustness the frequency selection procedure, all the subjects were offered the possibility to repeat *Step 2* and *Step 3*, the second time using as a control the two frequencies that were not selected during *Step 1*. In total, 8 subjects out of 20 agreed. These frequencies will be referred as secondary frequencies. These subjects were not informed of which frequency couple was indicated as the primary in *Step 1* and the two repetitions were executed in separate experimental sessions. The effects of the frequency selection on the 8 tested subjects are presented in Table 2 and Fig. 5 in which the average offline accuracy obtained with the frequency couples of both experimental sessions are compared. In order to quantitatively evaluate the effect of switching frequency couples, we subtract the average accuracy obtained with the primary frequencies by the average accuracy obtained with the secondary frequencies. The difference in the average accuracy between the two sets is then compared to the standard deviation of the two sessions. One subject (*s.18*) presented a clear reduction in accuracy with a decrease of 11.83% when imagining the primary frequencies. Four subjects (*s.8*, *s.19*, *s.7*, and *s.16*) did not show an appreciable difference, with a change respectively of −2.00%, −0.50%, +0.33%, and +3.83%. One subject (*s.11*) showed an apparent improvement, with an accuracy increase of 7.5% but still within the range of their standard deviation, which was 7.78% in their initial recording. Two subjects (*s.17* and *s.10*), instead, showed a clear improvement with an accuracy increasing of 9.67% and 15.33%.

**Table 2:** Comparison of the average accuracy for the frequency couple indicated by the frequency selection process and the other possible frequency couple as control, ordered following the difference between the average accuracy obtained with the primary and secondary frequencies.

	s.18	s.8	s.19	s.7	s.16	s.11	s.17	s.10
Primary frequencies (Hz)	(5 , 9)	(5 , 12)	(5 , 9)	(5 , 9)	(9 , 12)	(5 , 12)	(5 , 9)	(5 , 9)
Average accuracy (%)	58.33 ± 6.50	75.83 ± 6.72	57.67±7.65	55.00 ± 3.94	68.00 ± 8.29	57.17 ± 7.78	69.17 ± 6.92	48.33 ± 6.28
Secondary frequencies (Hz)	(7 , 12)	(7 , 9)	(7 , 12)	(7 , 12)	(5 , 7)	(7 , 9)	(7 , 12)	(7 , 12)
Average accuracy (%)	70.17 ± 8.04	77.83 ± 4.83	58.17±6.85	54.67 ± 6.14	64.17 ± 7.04	49.67 ± 3.40	59.50 ± 7.34	33.00 ± 7.26
Accuracy change (%)	−11.83	−2.00	−0.50	+0.33	+3.83	7.5	9.67	15.33

### BCI offline performance

**Fig. 6:**
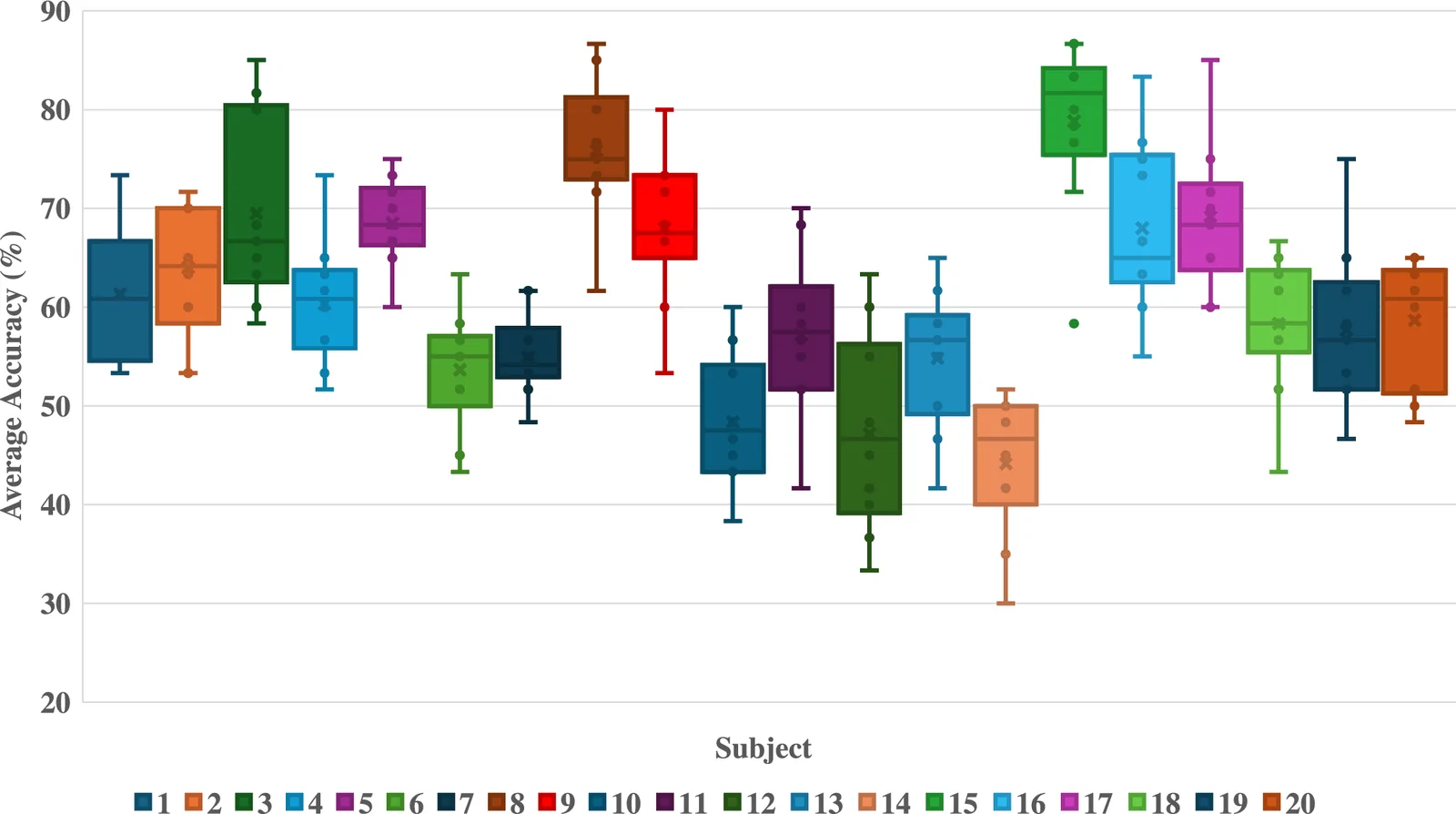
Box-plot representation of the overall accuracy for each subject evaluated separately for each different recording.

The VI data collected during the offline portion of the experiment was analyzed separately for each subject, as shown in Fig. 6. The average accuracy obtained with the trained SVM was compared to the confidence interval for a random classifier. Specifically, for a 3-classes random classifier with 160 recordings per class, a classification accuracy of 39.0% was reported by^47^ as the 99% confidence interval. Similarly, a value of 37.7% classification accuracy was reported as the 95% confidence interval for the same random classifier^47^. The offline experiments include 200 recordings per class, which are more than the 160 repetitions per class considered at most by Müller-Putz et al.^47^. We can then consider 39.0% of classification accuracy as a conservative estimation of the 99% confidence interval of a 3-classes random classifier with 200 recordings per class. Since the upper confidence bound for random classification decreases with the trial count, the actual 99% confidence interval for 200 will be lower than the considered 39.0%. For a normal distribution with the data reported in Müller-Putz et al.^47^, one could extrapolate the 99% confidence interval for the equivalent case with 200 repetitions per class, which would be of 38.4%. However, to obtain the precise confidence interval, the original study would have to be replicated and extended. For all 20 subjects, the average classification accuracy consistently exceeds the 99% confidence interval, as all subjects obtained an average classification accuracy higher than 39.00%. Observing separately the 10 recordings that the subjects executed during this portion of the experiment, presented in Fig. 6, most of the subjects obtained consistently a classification accuracy over the 99% confidence interval. Only three subjects (*s.10*, *s.12*, and *s.14*) had at least one recording with an accuracy lower than 39.00%, during the entire offline experiment.

### BCI online performance

**Fig. 7:**
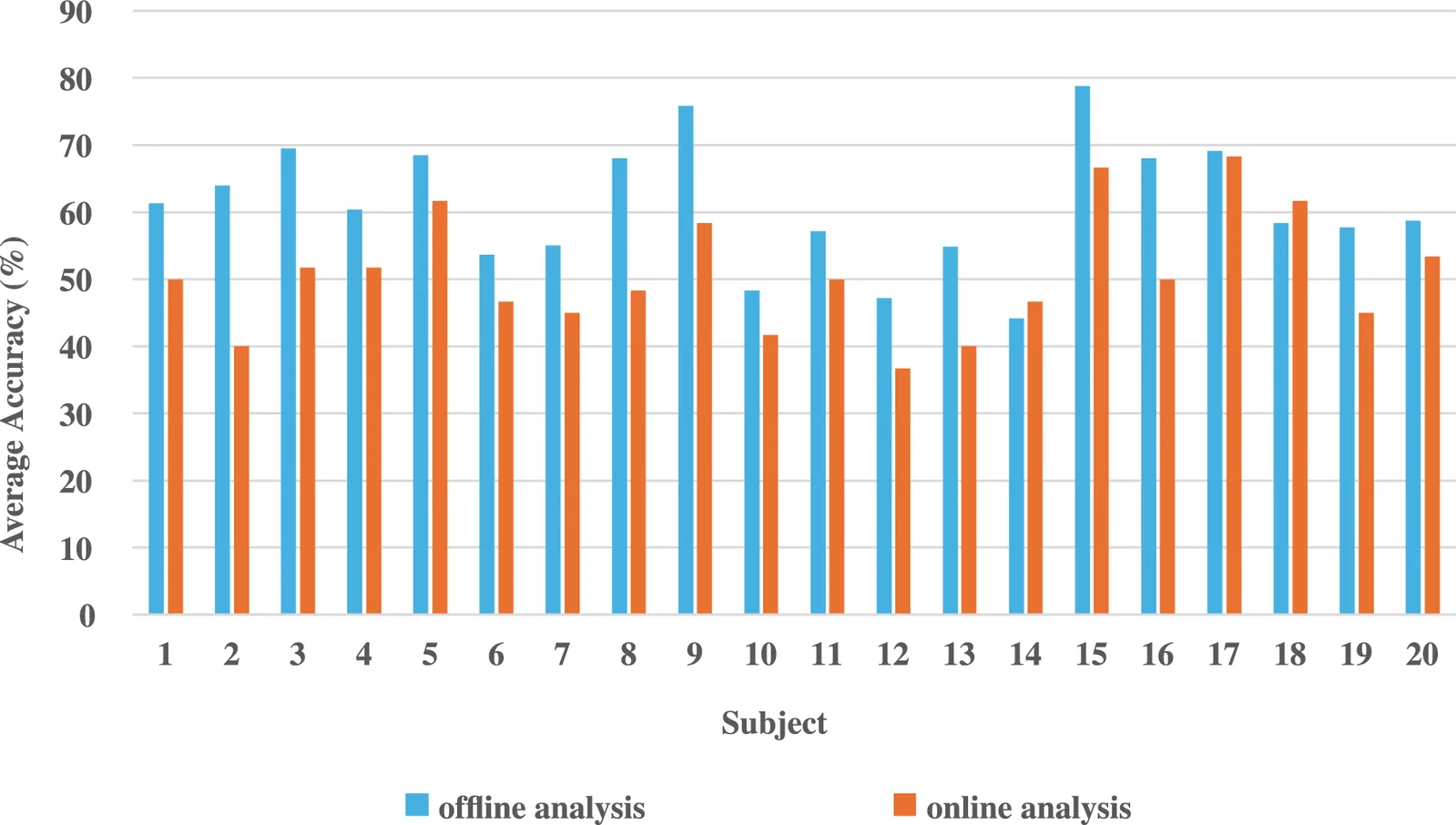
Comparison of the average accuracy obtained in the offline and the online experiment on all subjects.

For each subject, the classifier with the highest classification accuracy from the 5-fold cross-validation of *Step 3* was selected for the online experiment. As shown in Fig. 7, in most subjects there was a decrease in performance during the online task when compared to the offline measurements. As with the offline experiment, the results were compared to the confidence interval of a 3-classes random classifier with 20 repetitions for each class, for which the 99% and 95% confidence intervals for the random classifier corresponds to a classification accuracy of 50% and 45%, respectively^47^. Of the 20 subjects, 9 obtained an average classification accuracy greater than 50%, the 99% confidence interval, and 3 subjects obtained an average classification accuracy of precisely 50%. For the 95% confidence interval, out of the 20 subjects 16 had an average accuracy greater than 45% and 2 subjects had an average accuracy of 45%. We conducted a Welch’s t-test to confirm that the accuracy obtained during the online sessions is significantly different from a random chance distribution. The random distribution that was considered for this test has 20 repetitions for all 3 classes and therefore, as described by Müller-Putz et al.^47^, it has a standard deviation of 26.05. The Welch t-test was used to compare this random distribution and the recorded online classification data of all subjects which have a standard deviation of 8.62 and an average accuracy of 50.67 % (see Table 3). The t statistic obtained with the Welch’s t-test is 2.82 and the degrees of freedom of the system, obtained with the Welch-Satterthwaite equation, are 23.1. Thus, we can claim that the two distributions are significantly different, as the random distribution lies outside the 99% confidence interval of our online recordings.

**Fig. 8:**
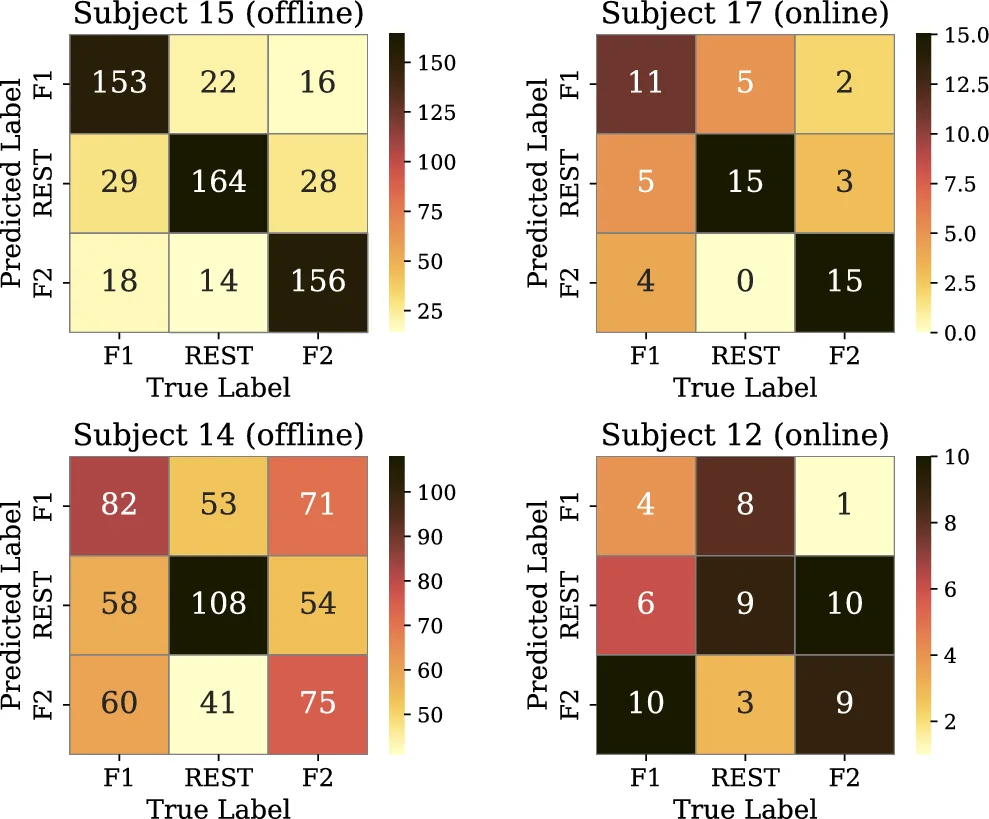
Confusion matrices for the subjects with the best and worst average accuracy in both the offline and online experiment.

The overall results are presented in Table 3. The confusion matrices for the four subjects that obtained the highest and the lowest average accuracy during the offline and the online experiment are presented in Fig. 8, to illustrate how the classification was distributed in these two extreme cases. The remaining confusion matrices are present in the supplementary material.

**Table 3:** Comparison between the offline and online average accuracy for each subject, with their corresponding frequency couple.

Subject	Frequency couple	Offline accuracy and Std Dev (%)	Online accuracy (%)
1	$(5\,\hbox {Hz}, 9\,\hbox {Hz})$	61.33 ± 6.36	50.00
2	$(5\,\hbox {Hz}, 9\,\hbox {Hz})$	64.00 ± 6.42	40.00
3	$(5\,\hbox {Hz}, 12\,\hbox {Hz})$	69.50 ± 8.88	51.67
4	$(5\,\hbox {Hz}, 7\,\hbox {Hz})$	60.33 ± 5.95	51.67
5	$(9\,\hbox {Hz}, 12\,\hbox {Hz})$	68.50 ± 4.11	61.67
6	$(5\,\hbox {Hz}, 9\,\hbox {Hz})$	53.67 ± 5.72	46.67
7	$(5\,\hbox {Hz}, 9\,\hbox {Hz})$	55.00 ± 3.94	45.00
8	$(5\,\hbox {Hz}, 12\,\hbox {Hz})$	75.83 ± 6.72	58.33
9	$(5\,\hbox {Hz}, 12\,\hbox {Hz})$	68.00 ± 7.06	48.33
10	$(5\,\hbox {Hz}, 9\,\hbox {Hz})$	48.33 ± 6.28	41.67
11	$(5\,\hbox {Hz}, 12\,\hbox {Hz})$	57.17 ± 7.78	50.00
12	$(5\,\hbox {Hz}, 9\,\hbox {Hz})$	47.17 ± 9.37	36.67
13	$(9\,\hbox {Hz}, 12\,\hbox {Hz})$	54.83 ± 6.60	40.00
14	$(5\,\hbox {Hz}, 7\,\hbox {Hz})$	44.17 ± 6.36	46.67
15	$(5\,\hbox {Hz}, 9\,\hbox {Hz})$	78.83 ± 8.13	66.67
16	$(9\,\hbox {Hz}, 12\,\hbox {Hz})$	68.00 ± 8.29	50.00
17	$(5\,\hbox {Hz}, 9\,\hbox {Hz})$	69.17 ± 6.92	68.33
18	$(5\,\hbox {Hz}, 9\,\hbox {Hz})$	58.33 ± 6.50	61.67
19	$(5\,\hbox {Hz}, 9\,\hbox {Hz})$	57.67 ± 7.65	45.00
20	$(5\,\hbox {Hz}, 9\,\hbox {Hz})$	58.67 ± 5.95	53.33

## Discussion

In this study we tested the operability of a VI-driven BCI system with an SVM classifier and devised a protocol for its use. The average accuracy across all subjects was 60.93% during the offline experiment and 50.67% during the online experiment. For the offline experiment the results were encouraging since all 20 subjects obtained an average accuracy over the 99% confidence interval (37.7% classification accuracy) for a random classifier with a comparable number of events per class^47^. Despite the fact that the subjects were only shown the actual visual stimuli at the start of the experiments during the training as described in Sect. 2 and did not see them again for the entirety of the experiment, the subjects were all able to achieve robust performance in the VI tasks. The subjects reported that they had fewer issues performing the task during the later sessions than during the initial ones, and did not report the onset of fatigue, or of any kind of headaches or similar discomforts due to the experiment.

During the online experiment, the average classification accuracy for all the subjects was still over the 99% confidence interval (50% in the conditions of the online experiment), but 3 subjects obtained an average accuracy of 50% and 9 obtained a lower average classification accuracy. When considering the decrease in accuracy between the offline and online experiment one should account or the fact that the online and offline session were structured in a similar but not identical way. Both experiments had the same 3 classes, however, while the offline recordings were comprised of a random sequence of events, with each event lasting 9 seconds, for a total of 9 minutes per recording, the online recordings had a fixed sequence of events, identical for all the subjects, with each one lasting only 5 seconds, resulting in 5 minutes for the entire recording. We believe that the decrease in performance is in part due to these differences as several of the subjects that showed a decrease in classification accuracy (such as s.2, s.3, s.8, s.9, and s.13) indicated after the recordings that the reduction in time was confusing after getting used to the longer tasks of the offline session.

Despite the globally positive experimental results, the frequency selection process did not prove as successful as initially hoped. A relevant accuracy increase was only reported in 2 of the 8 subjects. Even though for the overwhelming majority of subjects involved (7 of the 8) it was either beneficial or inconsequential, the number of subjects involved does not let us reach a definitive conclusion on the matter. It does however indicate that the classification accuracy is impacted by the frequencies used and that finding a better protocol that selects consistently the optimal frequencies for the subjects would beneficially impact the reliability of the system.

## Conclusion

We presented a BCI system that takes advantage of the characteristics of VI signals, opening novel applications to patients for which the use of standard visual stimuli is inconvenient. The system requires minimal training and equipment, can be easily used by subjects unfamiliar with imagery or other BCI techniques and the minimal setup does not require the supervision of expert medical personnel.

The results of the experiments we have conducted on 20 subjects show an average offline accuracy of 60.93%, and an average online accuracy of 50.67%, for three classes, which demonstrates that VI signal-based BCI applications are not only possible but also promising. Future works on this paradigm should aim at increasing the number of classes, which would improve the information transfer rate of the system.

## Supplementary Information

Supplementary Information.

## Data Availability

All the numerical data used for the statistical analysis of this paper is included in the additional material in form of the complete confusion matrices for all the subjects and all the experimental conditions.
