# Correction: A visual imagery paradigm for BCI strategies using imagined flickering patterns

**DOI:** 10.1038/s41598-026-67580-0

**Published:** 2026-08-21

**Authors:** Simone Priori, Paolo Ricci, Davide Consoli, Arturo Micheli, Adrien Merlini, Francesco P. Andriulli

**Affiliations:** 1 Department of Electronics and Telecommunication, Politecnico di Torino, 10129 Torino, Italy; 2 OpenGeoHub Foundation, 6865 HK Doorwerth, Netherlands; 3 Microwave Department, IMT Atlantique, 29238 Brest, France

Correction to: *Scientific Reports* 10.1038/s41598-026-41324-6, published online 04 March 2026

The original version of this Article contained errors in Table 3, where the data stated under the column ‘Frequency couple’ was incorrect for subjects 3, 5, 8, 9 and 11. The correct and incorrect values appear below.

Incorrect:

**Table:** 

Subject	Frequency couple
3	(5Hz, 9Hz)
5	(5Hz, 9Hz)
8	(9Hz,12Hz)
9	(9Hz,12Hz)
11	(9Hz,12Hz)

Correct:

**Table:** 

Subject	Frequency couple
3	(5 Hz,12 Hz)
5	(9 Hz,12 Hz)
8	(5 Hz,12 Hz)
9	(5 Hz,12 Hz)
11	(5 Hz,12 Hz)

The original Article has been corrected.

