# Supplementary material for: A visual imagery paradigm for BCI strategies using imagined flickering patterns
Source: Sci Rep. 2026 Mar 4;16:11967. doi: 10.1038/s41598-026-41324-6 (PMC13069113; doi:10.1038/s41598-026-41324-6)

Supplementary material—confusion matrices

This document contains the confusion matrices for the offline and online experiment of all subjects. In addition, for 8 of these subjects, the confusion matrices for the repetition of the offline experiments are presented.

Subject 1 (5Hz,9Hz):

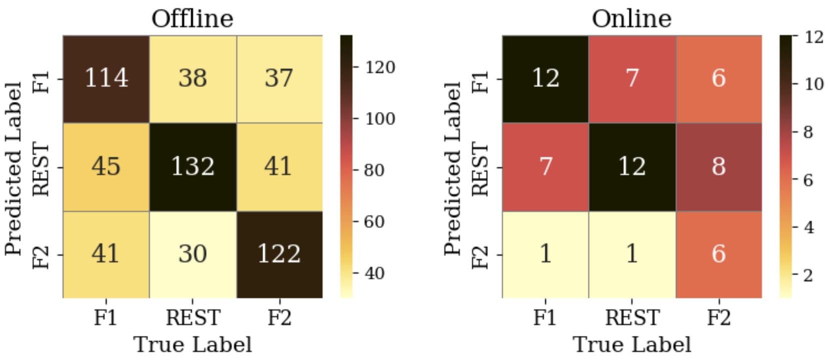

Subject 2 (5Hz,9Hz):

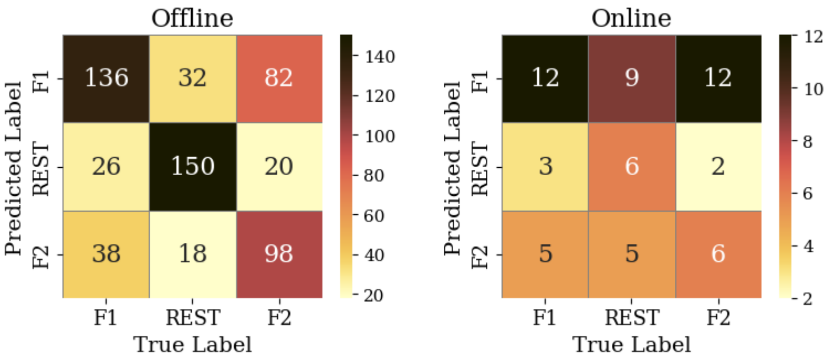

Subject 3 (5Hz,12Hz):

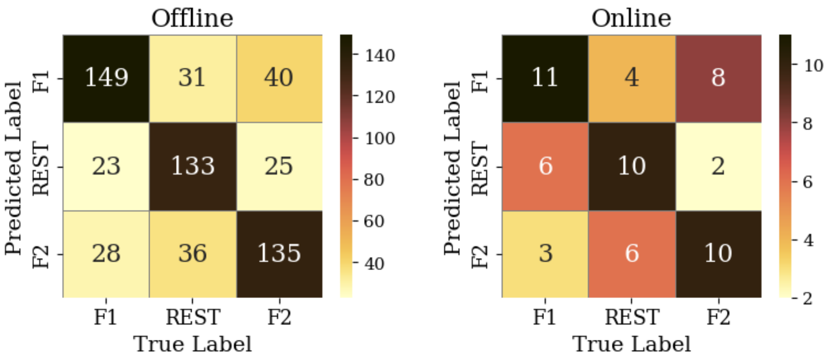

Subject 4 (5Hz,7Hz):

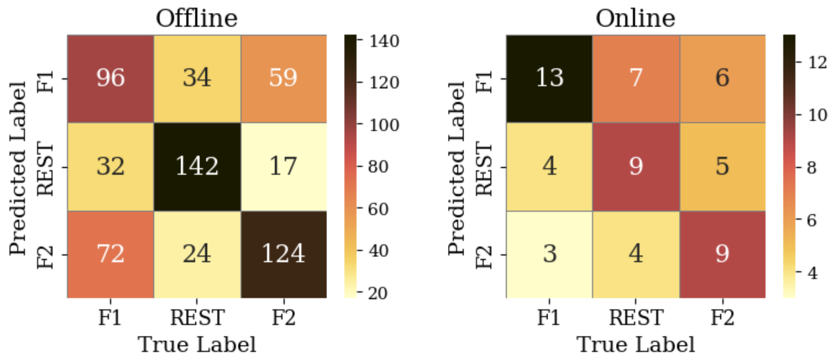

Subject 5 (9Hz,12Hz):

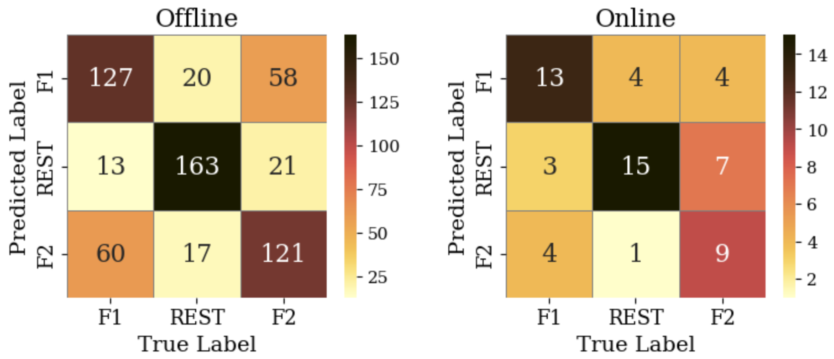

Subject 6 (5Hz,9Hz):

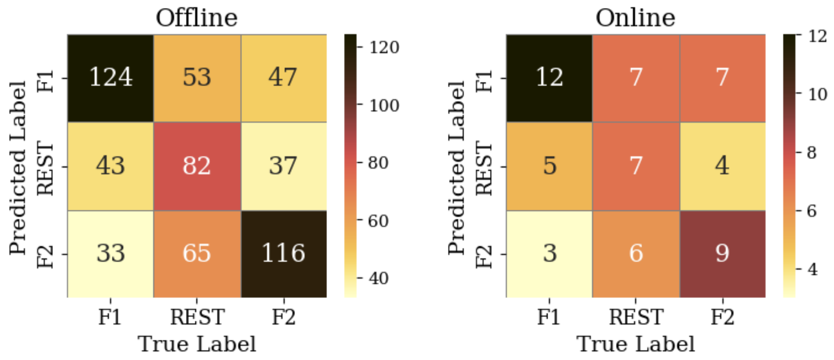

Subject 7 (5Hz,9Hz):

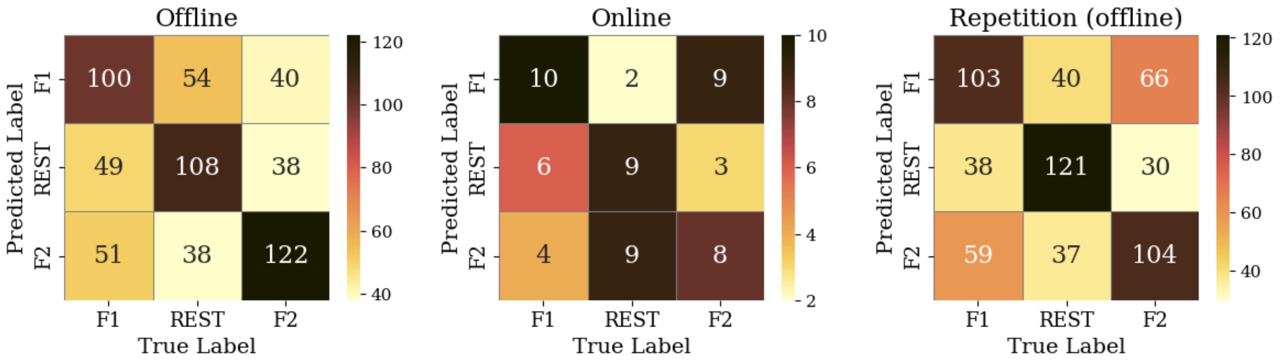

Subject 8 (5Hz,12Hz):

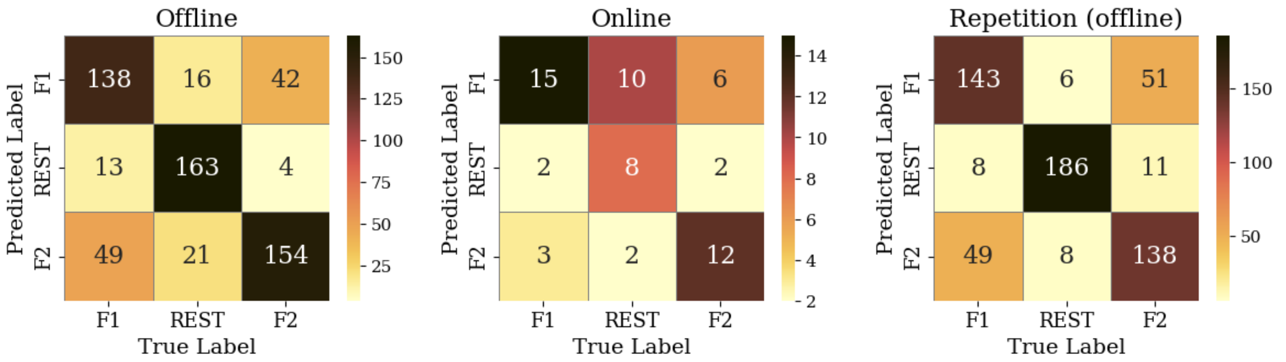

Subject 9 (5Hz,12Hz):

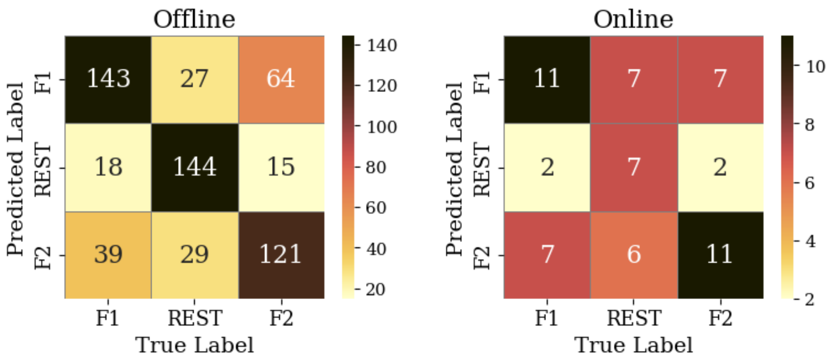

Subject 10 (5Hz,9Hz):

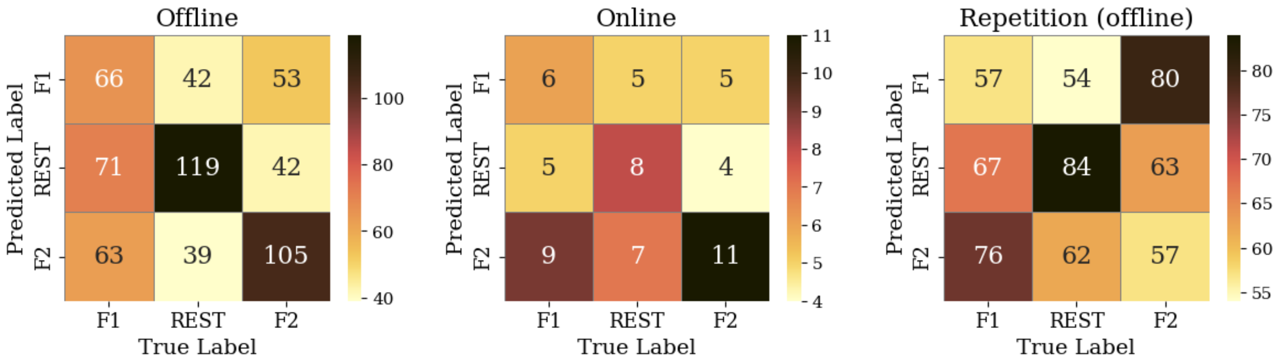

Subject 11 (5Hz,12Hz):

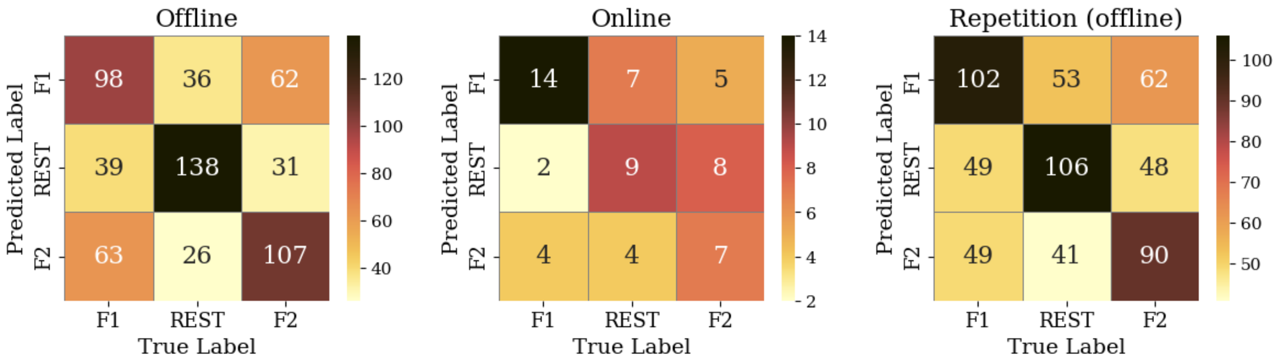

Subject 12 (5Hz,9Hz):

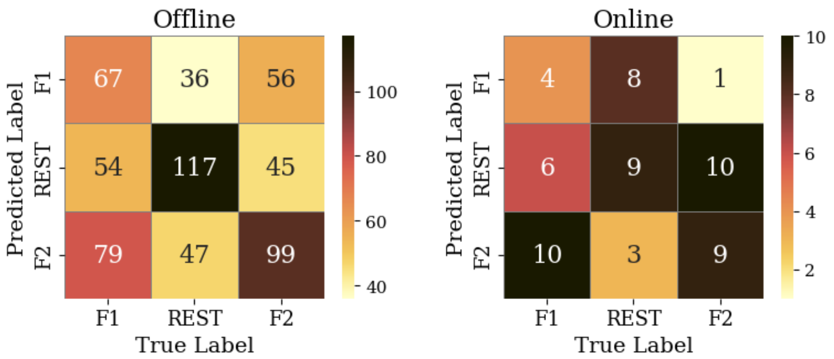

Subject 13 (9Hz,12Hz):

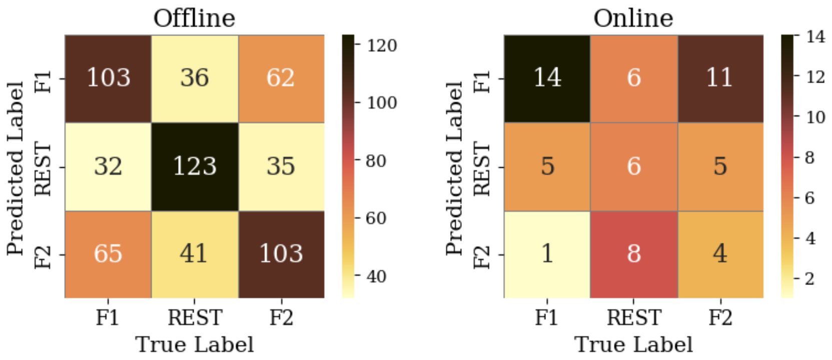

Subject 14 (5Hz,7Hz):

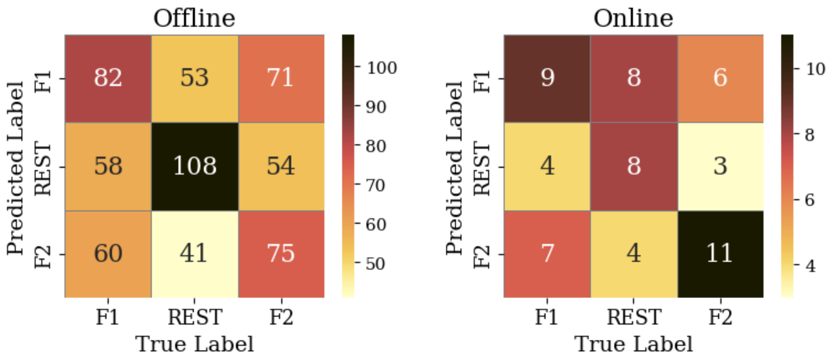

Subject 15 (5Hz,9Hz):

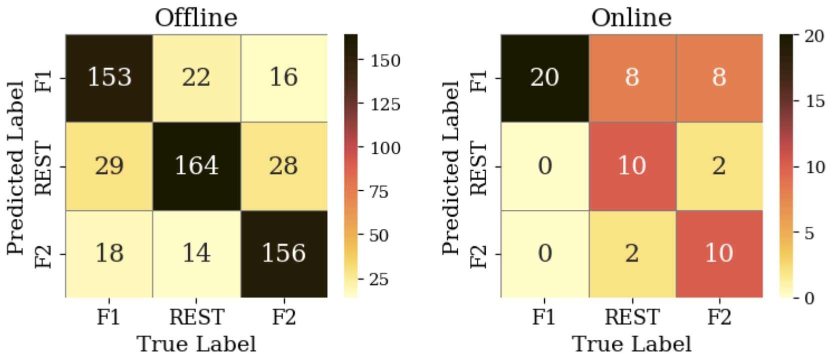

Subject 16 (9Hz,12Hz):

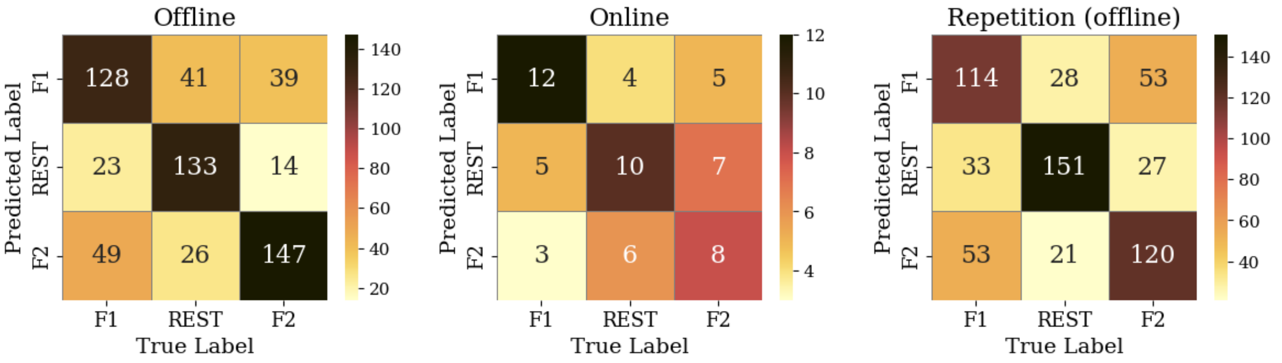

Subject 17 (5Hz,9Hz):

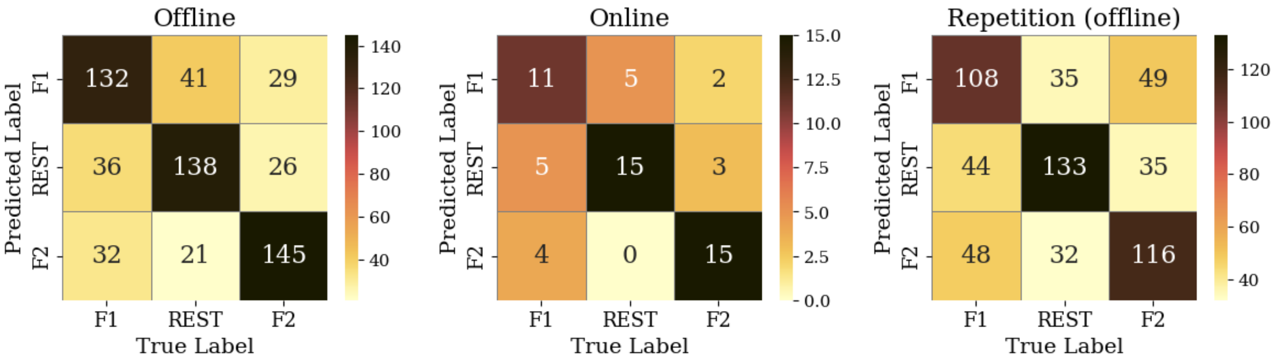

Subject 18 (5Hz,9Hz):

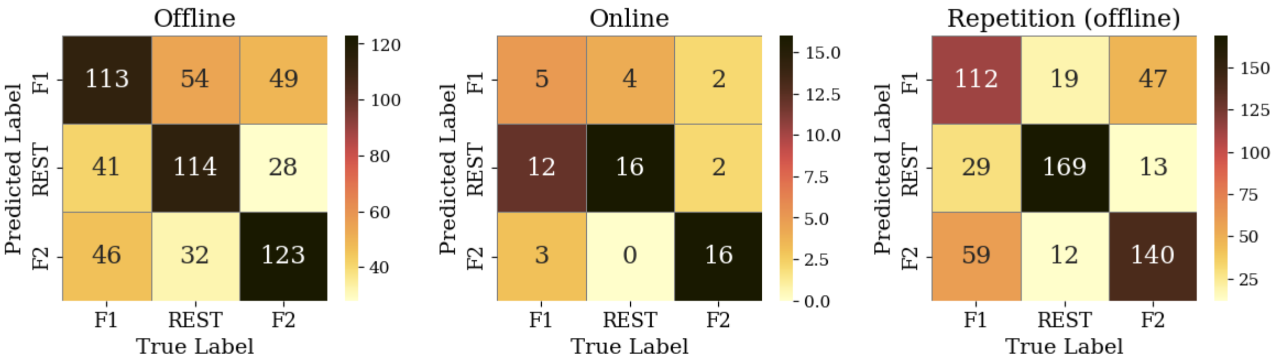

Subject 19 (5Hz,9Hz):

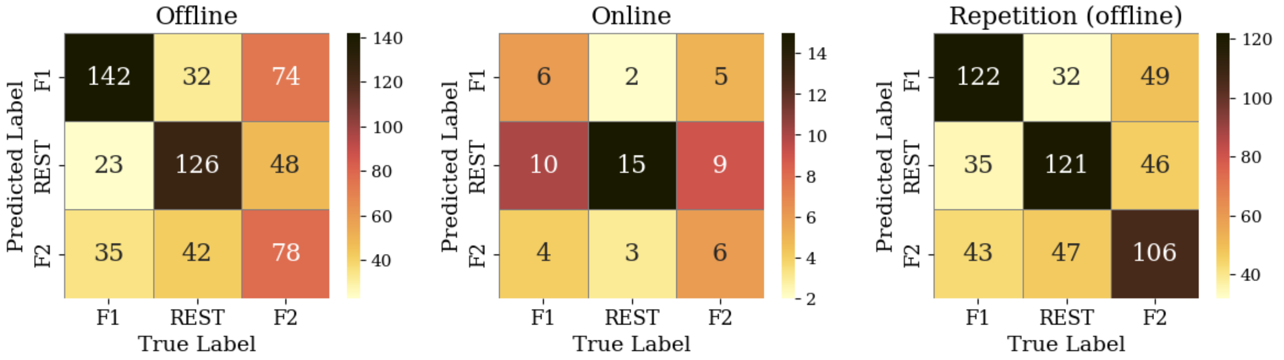

Subject 20 (5Hz,9Hz):

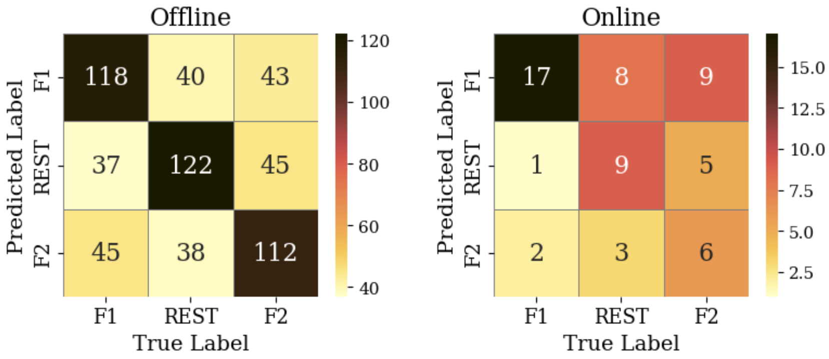

Supplement: Supplementary file 1 — Supplementary Information. [file 41598_2026_41324_MOESM1_ESM.pdf]
